# Cosmic and Thermodynamic Consequences of Kaniadakis Holographic Dark Energy in Brans–Dicke Gravity

**DOI:** 10.3390/e25040576

**Published:** 2023-03-27

**Authors:** Sania, Nadeem Azhar, Shamaila Rani, Abdul Jawad

**Affiliations:** 1 Department of Mathematics, COMSATS University Islamabad, Lahore Campus, Lahore 54000, Pakistan; sanianajeebullah98@gmail.com (S.); or drshamailarani@cuilahore.edu.pk (S.R.); 2 Department of Mathematics, National University of Modern Languages (NUML), Lahore Campus, Lahore 54000, Pakistan; nadeemazharsaeed@gmail.com

**Keywords:** Brane-Dicke theory, KHDE, cosmological parameters, thermodynamics

## Abstract

In this manuscript, we investigate the cosmological and thermodynamic aspects of the Brans–Dicke theory of gravity for a spatially flat FRW universe. We consider a theoretical model for interacting Kaniadakis holographic dark energy with the Hubble horizon as the infrared cutoff. We deal with two interaction scenarios ($Q_{1}$ and $Q_{2}$) between Kaniadakis holographic dark energy and matter. In this context, we study different possible aspects of cosmic evolution through some well-known cosmological parameters such as Hubble (H), deceleration (q), jerk (j), and equation of state $\left(\omega_{d}\right)$. For both interaction terms, it is observed that the deceleration parameter exhibits early deceleration to the current accelerating universe and also lies within the suggested range of Planck data. The equation of state parameter shows quintessence behavior (for the first interaction term) and phantom-like behavior (for the second interaction term) of the universe. The jerk parameter represents consistency with the ΛCDM model for both interaction terms. In the end, we check the thermodynamic behavior of the underlying model. It is interesting to mention here that the generalized second law of thermodynamics holds for both cases of interaction terms.

## 1. Introduction

The most mysterious and persistent problem in today’s cosmology is the current accelerated expansion of the universe. It is widely recognized that the universe underwent a late-time transition from the epoch of matter to the phase of accelerated expansion. Well-known observational data, such as gravitational lensing [1], Ia supernovae [2,3] and cosmic microwave background radiation [4,5], provide authentic evidence that we are in this transition phase. Dark energy (DE) [6,7,8] plays a prominent role in this expansion due to its negative pressure. The first candidate to describe this repulsive force is a cosmological constant, whose equation of state parameter is -1. Unfortunately, this simplest candidate has faced well-known problems, such as fine-tuning and the cosmological constant problems [9,10]. Therefore, researchers are trying to identify the candidate for DE that not only explains the current accelerated expansion of the universe but also addresses the above-mentioned problems.

The theory of dark energy (DE) with negative pressure has received much recognition among the existing theories that attempt to explain the accelerated expansion of the universe. There are two basic ways to account for the universe’s late-time accelerated expansion. The first choice is the dynamical DE theories, which are proposed by modifying the matter part of the Einstein-Hilbert action. Several dynamic DE models have been proposed, including quintessence [11,12], *k*-essence [13,14], phantom [15,16], Chaplygin gas [17,18] and holographic DE (HDE) [19]. These models are designed to explore the nature of DE and resolve issues such as cosmic coincidence and fine-tuning. A second approach is alternative theories of gravity that are obtained from the geometrical part of the Einstein-Hilbert action and include some extra degrees of freedom that can drive the universe’s accelerated expansion [20,21,22,23,24,25]. The basic alternative theories of gravity include f(R) (where *R* denotes the Ricci scalar) [26], f(T) (*T* denotes the torsion scalar) [27], f(G) (*G* is the Gauss-Bonnet term) [28], Dynamical Chern-Simons Modified Gravity [29], Brans–Dicke (BD) theory [30,31], and others.

The BD theory (more generally the scalar-tensor theory) is considered the best candidate, alternative to general relativity. In this natural modification, the non-minimal coupled scalar field is replaced by Newtons gravitational constant [32,33]. This theory addressed major cosmological issues such as inflation, quintessence and the cosmic expansion of the universe. Well-known braneworld scenario has been investigated in the context of this higher dimensional theory. Moreover, BD theory concerns the geometrical nature of the scalar field which is considered an attractive feature of this theory. In this BD theory with a chameleon scalar field, the scalar potential and the matter field emerge from the geometry of the extra dimensions rather than some ad hoc assumptions, is an appropriate and fundamental alternative to the standard BD theory [34,35,36,37,38]. Wesson and Ponce de Leon [39] induced matter theory, where contends that the additional parametric terms resulting from the added dimension Einstein’s 5D equations can also be connected with an efficient energy tensor in 4D theory was inspired by this disparity between a fine marble of geometrical as well as the low-grade wood of matter. They observed that the 4D non-vacuum universe is similar to the 5D vacuum universe, geometrically seen by [39]. By using the induced matter theory, a modified BD theory is defined as a 4D BD theory with a self-interacting potential and an efficient matter field.

Further, Rasouli et al. [40] also established a modified BD (MBDT) theory in which the matter and and potential are entirely emerge from geometry rather than being introduced into the action via ad hoc assumption. In [41], authors investigated the cosmological aspects of Barrow HDE in the framework of BD cosmology. They Considered both interaction and non-interaction scenarios and checked the stability analysis. They found the consistency of the underlying model. Amani and Halpern [42] checked the energy conditions in a modified BD gravity. Ghaffari [43] studied the cosmological consequences of non-interaction Kaniadakis HDE (KHDE) in the context of BD gravity. He found some cosmological parameters and obtained the ranges that are compatible with observations.

Among several DE models, the HDE model and its modified families as Tsallis HDE [44], Rényi HDE [45] and Barrow HDE [46] are used in literature to illustrate how the cosmos is expanding. Initially, Li [19] developed the energy density of HDE model to describe the accelerated expansion, which is driven with help of the holographic principle [18,47,48,49]. The HDE model is a viable method for resolving DE issues. The holographic theory is based on the system that expresses the scale for a system’s number of degrees of freedom is primarily determined by the area that was taken into consideration. The interesting cosmic implications of HDE are also confirmed to be in line with observations [50,51,52,53]. The basic concept for the formation of HDE is the relationship between the entropy of the system and geometric factors such as its radius. The common entropy is the Bekenstein-Hawking entropy, which is used in cosmology and black holes and is obtained from the fundamental Boltzmann-Gibbs entropy.

The Boltzmann-Gibbs entropy, however, has been generalized by Kaniadakis into a one-parameter concept known as Kaniadakis entropy [54,55]. It is the consequence of a coherent and self-consistent dynamical statistical theory, that maintains the fundamental characteristics of conventional statistical principles. The distribution functions in this expanded statistical theory is a one-parameter continuous distortion of the standard Maxwell-Boltzmann models. The arguments in [56] led us to apply the HDE structure to the Kaniadakis problem from the perspective of complex quintessence and the BD theory. In [57] authors used the holographic fundamentals with Kaniadakis entropy and developed the energy density of KHDE model. This energy density for Hubble horizon as infrared cutoff is given as [58]

(1) $$\begin{array}{c} \rho_{d}=3c^{2}\phi^{2}H^{2}+\frac{u^{2}\phi^{2}}{H^{2}}, \end{array}$$

where $H=\frac{\dot{a}}{a}$ is the Hubble parameter (where *a* denotes the scale factor and “.” expresses the derivative w.r.t time *t*), *c* denotes the model parameter and *u* is the Kaniadakis entropy parameter that is defined as −1<u<1. Furthermore, we consider the relation $\phi \propto a^{n}$ [59]. From this relation, we can obtained the following expressions

(2) $$\begin{array}{ccc} \dot{\phi } & = & n\phi H, \end{array}$$

(3) $$\begin{array}{ccc} \ddot{\phi } & = & H^{2}n^{2}\phi +\phi n\dot{H}. \end{array}$$

Few early attempts in the literature regarding this framework [60,61] the models that came out of those efforts were interesting. The authors choose Hubble horizon rather than a future event horizon in a fundamental holographic representation was a different experiment. The goal and inspiration for this study is to assess the existing view of the cosmos using cosmological and thermodynamic methods about to with concerning the BD and the BD theory with a chameleon scalar field, utilizing the KHDE model.

This paper is organized as follows: In Section 2, we discuss the interaction between the dark sectors in the contexts of the standard BD theory and the BD theory with a chameleon scalar field Or Chameleon BD theory. In Section 3, we describe the effects of the $Q_{1}$ interaction term about to with concerning the standard BD and the BD theory with a chameleon scalar field and determine a few cosmological parameters. Similarly, in Section 4, we analyze the effects of a $Q_{2}$ interaction term to discuss the cosmography in the context of BD and the BD theory with a chameleon scalar field. In Section 5, we examine the thermodynamics with interaction terms $Q_{1}$ and $Q_{2}$ in the context of Kaniadakis entropy. In Section 6, we conclude our findings.

## 2. Basic Formalism

In this section, we discuss the basic equations of standard BD theory and the BD theory with a chameleon scalar field.

### 2.1. Standard Brans–Dicke Theory of Gravity

Now, we discuss one of the viable theories of gravity named BD theory of gravity. In the BD theory gravitational constant (G) is replaced with the BD scalar field φ $\left(G\left(\varphi \right)=\frac{1}{\varphi }\right)$. Before starting the DB action, first we consider non-flat Friedmann-Robertson-Walker (FRW) universe, which is given as

(4) $$\begin{array}{c} ds^{2}=dt^{2}-a^{2}\left(t\right)\left(\frac{dr^{2}}{1-kr^{2}}+r^{2}d\theta^{2}+r^{2}sin^{2}\theta d\phi^{2}\right), \end{array}$$

where the curvature of the space is represented by *k* with k=−1, 0 and 1 refers to open, flat and closed universe. The action for the BD theory of gravity in the Jordan frame is defined as [30]

(5) $$\begin{array}{c} S=\int \sqrt{g}\left[-\varphi R+\omega \frac{g^{\alpha \beta }\varphi_{,\alpha }\varphi_{,\beta }}{\varphi }+L_{m}\right]d^{4}x, \end{array}$$

where *R* is the Ricci scalar, ω and φ stand for the BD coupling parameter and the BD scalar field, respectively. $L_{m}$ denotes the Lagrangian for matter fields, which does not depend on the scalar field. The above action can be transformed $\varphi \to \frac{\phi^{2}}{8\omega }$, where the scalar field φ is redefined by a new scalar field ϕ. In such a way, the new action in canonical form is obtained as [32]

(6) $$\begin{array}{c} S=\int \sqrt{g}\left[-\frac{\phi^{2}}{8\omega }R+\frac{g^{\alpha \beta }\phi_{,\alpha }\phi_{,\beta }}{2}+L_{m}\right]d^{4}x, \end{array}$$

the above action is obtained by replacing non-minimal coupling term $\phi^{2}R$ instead of the Einstein-Hilbert term $\frac{R}{G}$ (where $G^{-1}=\frac{2\pi \phi^{2}}{\omega }$) and ϕ is defined as the power law of a scale factor $\left(\phi \propto a^{n}\right)$. The acceleration of the cosmos at the current time cannot be described by either the basic theory of general relativity or the BD theory without the inclusion of a cosmic constant term or another source term functioning similarly into the field equations [62,63]. Varying the action (6) (for flat FRW spacetime) with respect to (w.r.t) metric tensor by considering the universe filled with dust and KHDE, one can obtain the field equations for as follow [64]

(7) $$\begin{array}{c} \frac{3\phi^{2}H^{2}}{4\omega }+\frac{3H\phi \dot{\phi }}{2\omega }-\frac{{\dot{\phi }}^{2}}{2}=\rho_{d}+\rho_{dm}, \end{array}$$

(8) $$\begin{array}{c} -\frac{\phi^{2}}{4\omega }\left(\frac{2\ddot{a}}{a}+H^{2}\right)-\frac{H\phi \dot{\phi }}{\omega }-\frac{\phi \ddot{\phi }}{2\omega }-\frac{{\dot{\phi }}^{2}}{2}\left(1+\frac{1}{\omega }\right)=P_{d}, \end{array}$$

(9) $$\begin{array}{c} \ddot{\phi }+3H\dot{\phi }-\frac{3}{2\omega }\left(\frac{\ddot{a}}{a}+H^{2}\right)\phi =0, \end{array}$$

where $\rho_{d}$ and $\rho_{dm}$ are the densities of DE and dark matter (DM) while $P_{d}$ expresses the pressure of DM. In this work, we consider the KHDE as the energy density of the universe. Equation (7) can also be written in form of fractional energy density by defining the critical density $\frac{3\phi^{2}H^{2}}{4\omega }$. Hence, Equation (7) can be written for flat FRW universe as

(10) $$\begin{array}{c} \Omega_{d}+\Omega_{dm}=1+\Omega_{\phi }, \end{array}$$

where the dimensionless density parameters are presented by $\Omega_{dm}=\frac{4\omega \rho_{dm}}{3\phi^{2}H^{2}},\;\Omega_{d}=\frac{4\omega \rho_{d}}{3\phi^{2}H^{2}},\;\Omega_{\phi }=2n\left(\frac{n\omega }{3}-1\right)$.

### 2.2. The BD Theory with a Chameleon Scalar Field

Let us consider the BransDicke action with a chameleon scalar field [65]

(11) $$\begin{array}{c} S=\int \left(f\left(\phi \right)L_{m}-\frac{\phi R}{2}+V\left(\phi \right)+\frac{\omega }{2\phi }g^{\alpha \beta }\phi_{,\alpha }\phi_{,\beta }\right)\sqrt{g}d^{4}x, \end{array}$$

where V(ϕ) is a scalar field potential, f(ϕ) denotes an arbitrary function of ϕ. If we replace f(ϕ)=1= constant, then it gives the usual BD theory [65]. From the variation of Equation (11) and by using the metric tensor $g_{\mu \nu }$ and ϕ the following field equations are obtained as

(12) $$\begin{array}{ccc} & & \phi G_{\mu \nu }=T_{\mu \nu }^{\phi }+f\left(\phi \right)T_{\mu \nu }^{m}, \end{array}$$

(13) $$\begin{array}{ccc} & & \left(2\omega +3\right)\nabla^{\mu }\nabla_{\mu }\phi +2\left(2V-V^{\prime }\phi \right)=T^{m}f-2f^{\prime }\phi_{m}, \end{array}$$

where $G_{\mu \nu }$ denotes the Einstein tensor, $T^{m}=g^{\mu \nu }T_{\mu \nu }^{m}$, $V^{\prime }=\frac{dV}{d\phi }$ and $f^{\prime }=\frac{df}{d\phi }$. In Equation (12), $T_{\mu }^{\phi }\nu$ and $T_{\mu }^{\left(m\right)}\nu$ and were defined as

(14) $$\begin{array}{ccc} T_{\mu \nu }^{\phi } & = & \frac{\omega }{\phi }\left(\nabla_{\mu }\phi \nabla_{\nu }\phi -\frac{1}{2}g_{\mu \nu }\nabla_{\alpha }\phi \nabla^{\alpha }\phi \right)-V\left(\phi \right)g_{\mu \nu }+(\nabla_{\mu }\nabla_{\nu }\phi \\ & - & g_{\mu \nu }\nabla_{\mu }\nabla^{\mu }\phi ), \end{array}$$

(15) $$\begin{array}{ccc} T_{\mu \nu }^{m} & = & \frac{-2\delta (L_{m}\sqrt{-g})}{\sqrt{-g}\left(\delta g^{\mu \nu }\right)}. \end{array}$$

Hence, we have the following field equation for flat FRW metric

(16) $$\begin{array}{ccc} 3H^{2} & = & \frac{f}{\phi }\rho +\frac{\omega \dot{\phi^{2}}}{2\phi^{2}}-3H\frac{\dot{\phi }}{\phi }+\frac{V}{\phi }, \end{array}$$

here $\rho =\rho_{dm}+\rho_{d}$. In this paper, we consider $V=V_{0}\phi^{\beta }$ and $f=f_{0}\phi^{\gamma }$ [66] where $V_{0}$, $f_{0}$, β and γ are constants.

## 3. Interaction between Dark Sector

It has been observed [67] that most of the universe contained DM and DE. The DE is responsible for the accelerated expansion universe while DM is the hypothetical form of matter which cannot absorb, reflect or emit light. One cannot see it directly but observe its effects through gravitational attraction. Well-known observational data suggested that DE and DM occupied 68.3% and 26.8% respectively of our universe and the remaining 4.9% consists of regular matter that is seen by the human eye. The formation of a structure is effected due to the interaction between DE and DM at distinct scales and times. The cosmic coincidence problem is considered as a well-known puzzle within this perspective. This problem is elaborated that the densities of DM and DE are of the same order of magnitude, given that they evolve very differently with redshift? One can alleviate this problem through appropriate coupling between the DM and DE. This coupling is justified from a phenomenological point of view, or give a covariant prescription for it and then let the data be the judge of its viability. In this scenario, the energy conservation equations become as

(17) $$\begin{array}{ccc} {\dot{\rho }}_{d}+3H\rho_{d}\left(1+\omega_{d}\right) & = & -Q, \end{array}$$

(18) $$\begin{array}{ccc} {\dot{\rho }}_{dm}+3H\rho_{dm} & = & Q, \end{array}$$

where $\omega_{d}$ is the EoS parameter and *Q* is the interaction term. This interaction term plays a very important term as it expresses the rate of energy exchange between DE and DM. It is interesting to mention here that if Q>0 shows the energy exchange from KHDE to DM while if Q<0 leads to an energy transfer from DM to KHDE. If Q=0, this corresponds to non-interaction scenario. The interaction scenario is considered a more general and comprehensive way to investigate cosmic evolution. Various linear and non-linear interaction terms have been proposed in literature [68]. These interaction terms are the function of $\rho_{d}$, $\rho_{dm}$, *H* and their linear combinations. In this work, we discuss two types of interaction terms that are defined as follow [69,70,71,72,73,74,75]

(19) $$\begin{array}{ccc} Q_{1} & = & 3Hb\rho_{d}+\hat{\gamma }{\dot{\rho }}_{d}, \end{array}$$

(20) $$\begin{array}{ccc} Q_{2} & = & 3Hb\rho_{dm}+\hat{\gamma }{\dot{\rho }}_{dm}. \end{array}$$

In these interactions terms *b* and $\hat{\gamma }$ are constants and bounded between −1≤b≤1 and $-1\leq \hat{\gamma }\leq 1$. These kinds for the interaction terms have a minimal impact on the evolution of the universe’s overall energy budget. In fact, these terms may be understood as a first order Taylor expansion. Furthermore, they are predicated on the idea that the dark particle propagator depends on energy. These interactions have been proven to be a useful method for resolving the coincidence issue [76].

## 4. $Q_{1}$ Interaction Term

Furthermore, we consider the interaction $\left(Q_{1}\right)$ and investigate the cosmic evolution of the KHDE model for both standard BD and the BD theory with a chameleon scalar field.

### 4.1. For Standard BD Theory

In this section, we study the global dynamics of the universe through some very important cosmological parameters in the framework of Standard BD theory. Hence, we find the Hubble parameter, deceleration parameter, equation of state (EoS) parameter and jerk parameter by using the KHDE model.

**Figure 1:**
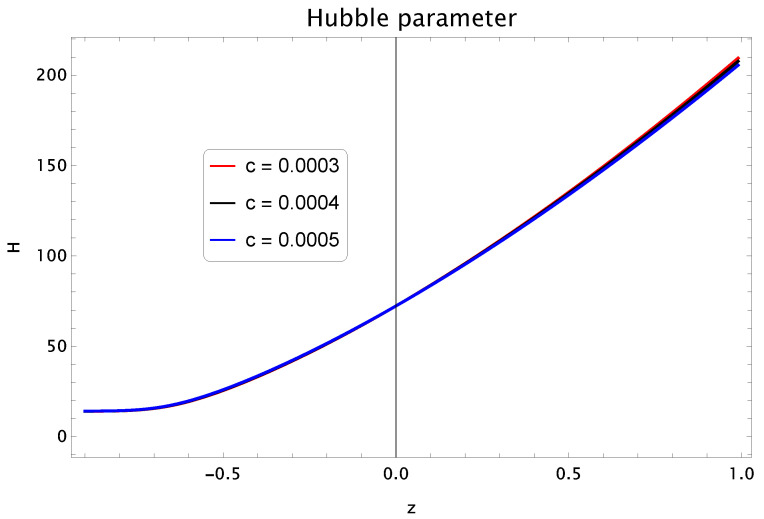
Plot of *H* versus *z* by choosing three different values of *c* for interacting KHDE model in the standard BD theory.

**Figure 2:**
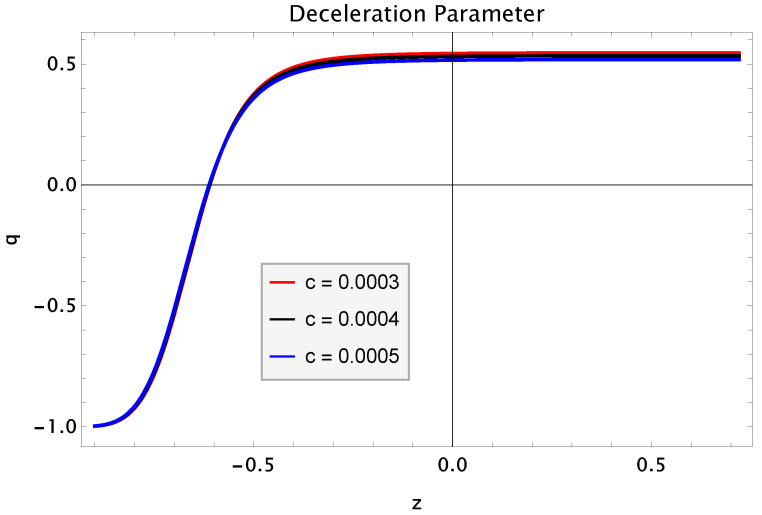
Plot of *q* against *z* for interacting KHDE model in the standard BD theory.

**Figure 3:**
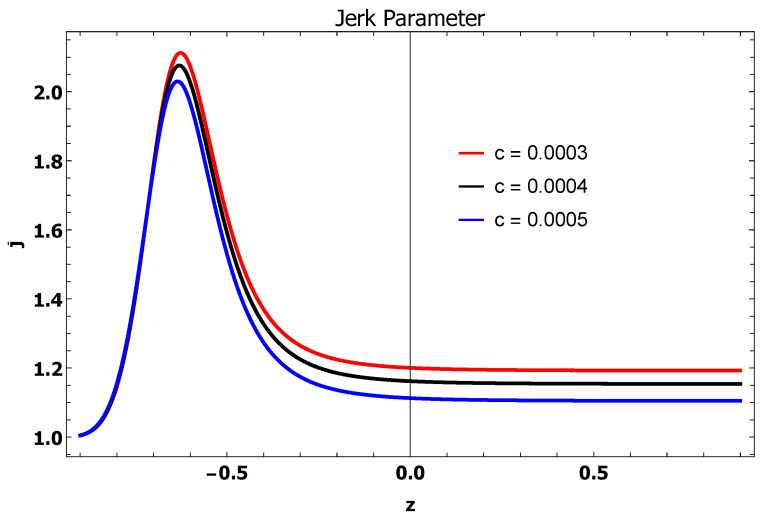
Plot of jerk parameter *j* against redshift parameter *z* for interacting KHDE model in the standard BD theory.

**Figure 4:**
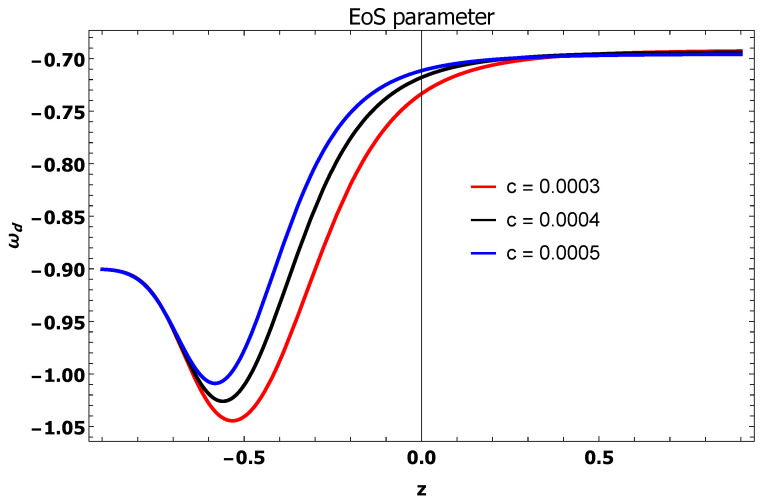
Graphical presentation of EoS parameter $\omega_{d}$ plot against *z* for interacting KHDE model in the standard BD theory.

- **Hubble Parameter:** This parameter sets the scale of our universe at present time. The Hubble constant $\left(H_{0}\right)$ is called the current value of Hubble parameter which lies in the range 65–75 km/s/Mpc. Now, taking the derivative of Equation (7) w.r.t cosmic time and by substituting the values of $\dot{\phi }$, $\ddot{\phi }$, $\dot{\rho_{d}}$ and ${\dot{\rho }}_{dm}$, we obtain
  (21) $$\begin{array}{c} \frac{\dot{H}}{H^{2}}=\frac{\Omega_{d}\left(9b+6n\gamma +9+6n\right)-\left(9+6n\right)\left(\frac{2n}{3}\left(n\omega -3\right)+1\right)}{6+12n-4\omega n^{2}-24\omega c^{2}+2\left(3\Omega_{d}-12\omega c^{2}\right)-24\omega \gamma c^{2}+2\gamma \left(3\Omega_{d}-12\omega c^{2}\right)}. \end{array}$$
  Next, we convert the above expression of Hubble parameter in term of redshift parameter *z*. This relation is defined as
  (22) $$\begin{array}{c} H^{\prime }=\frac{-\dot{H}}{H(1+z)}. \end{array}$$
  where $H^{\prime }=\frac{dH}{dz}$. Using above expression, Equation (21) become
  (23) $$\begin{array}{c} H^{\prime }=\frac{\left(-H\right)(\Omega_{d}\left(9b+6n\gamma +9+6n\right)-\left(9+6n\right)\left(\frac{2n}{3}\left(n\omega -3\right)+1\right))}{\left(1+z\right)(6+12n-4\omega n^{2}-24\omega c^{2}+2\left(3\Omega_{d}-12\omega c^{2}\right)-24\omega \gamma c^{2}+2\gamma \left(3\Omega_{d}-12\omega c^{2}\right))}. \end{array}$$
  The Hubble parameter *H* versus redshift function *z* by selecting H(z=0)=72.3 is plotted in the Figure 1. We take three different values of c=0.0003, 0.0004, 0.0005 and b=−0.1. This plot is showing that the current value of Hubble parameter $H_{0}=74$ which is compatible with observational bounds [77]. Additionally, the range of this parameter lies in H=107.5±92.5.

- **Deceleration Parameter:** This important parameter is denoted by *q*. It differentiates the decelerated as well as the accelerated phase of the universe. The mathematical form of this parameter as the function of the Hubble parameter is given as follows
  (24) $$\begin{array}{c} q=-\frac{\ddot{a}}{aH^{2}}=-1-\frac{\dot{H}}{H^{2}}. \end{array}$$
  The different phases of the universe corresponding to different values of this parameter is defined as follows
  (25) $$\begin{array}{c} q=\left\{\begin{array}{cc} \mathrm{Decelerated expansion} & \;\mathrm{if}\;\;q>0,\\ \mathrm{Constant expansion} & \;\;\;\;\mathrm{if}\;\;\;\;q=0,\\ \mathrm{Acceleration with power law expansion} & \;\mathrm{if}\;\;-1<q<0,\\ \mathrm{Exponential expansion} & \;\mathrm{if}\;\;q=-1,\\ \mathrm{Expension is super exponential} & \;\mathrm{if}\;\;q<-1. \end{array}\right. \end{array}$$
  We obtain the expression of *q* by inserting the value of $\frac{\dot{H}}{H^{2}}$ from Equation (21) into Equation (24) as
  (26) $$\begin{array}{c} q=-1-\frac{\Omega_{d}\left(9b+6n\gamma +9+6n\right)-\left(9+6n\right)\left(\frac{2n}{3}\left(n\omega -3\right)+1\right)}{6+12n-4\omega n^{2}-24\omega c^{2}+2\left(3\Omega_{d}-12\omega c^{2}\right)-24\omega \gamma c^{2}+2\gamma \left(3\Omega_{d}-12\omega c^{2}\right)}. \end{array}$$
  In Figure 2, the deceleration parameter *q* is plotted against redshift *z* for the three different values of *c*. It has been noted that the universe is showing the early decelerated phase to current accelerated phase. For z>−0.6, the trajectories show the decelerated phase while z<−0.6 these trajectories indicate the accelerated expansion of the universe [77].

- **Jerk Parameter:** This parameter provides a convenient and alternative way to describe cosmological methods close to concordance ΛCDM model. If j=1 (constant) it corresponds to ΛCDM model. Mathematically, this parameter is defined as the third derivative of a scale factor w.r.t time *t* as [78,79,80]
  (27) $$\begin{array}{c} j=\frac{\frac{d^{3}a}{dt^{3}}}{aH^{3}}=q\left(2q+1\right)+\frac{dq}{dz}\left(1+z\right). \end{array}$$
  Using Equation (26) in Equation (27), it results
  (28) $$\begin{array}{ccc} j & = & (-1-\left[\Omega_{d}\left(9b+6n\gamma +9+6n\right)-\left(9+6n\right)\left(\frac{2n}{3}\left(n\omega -3\right)+1\right)\right]\\ & \times & [6+12n-4\omega n^{2}-24\omega c^{2}+2\left(3\Omega_{d}-12\omega c^{2}\right)-24\omega \gamma c^{2}+2\gamma (3\Omega_{d}\\ & - & 12\omega c^{2}){]}^{-1})\times \left(2q+1\right)+\frac{dq}{dz}\left(1+z\right). \end{array}$$
  Figure 3 represents the plot of a jerk parameter *j* against the redshift *z*. From the graph, it can be seen that all the trajectories of the jerk parameter show positive behavior in the past, present and future era and all the trajectories converge to 1 which corresponds to ΛCDM model.

- **Equation of State Parameter:** This parameter is denoted by $\omega_{d}$ is defined as the ratio of pressure to DE $\omega_{d}=\frac{p}{\rho }$. The EoS parameter is a powerful tool to define the accelerated and decelerated phases of the universe. Its different features related to phase transition are given in the following table:
Accelerated Phase$-1<\omega_{d}<-\frac{1}{3}$Quintessence$\omega_{d}=-1$Cosmological Constant$\omega_{d}<-1$Phantom-Dominated EraDecelerated Phase$\omega_{d}=1$Stiff Fluid$\omega_{d}=\frac{1}{3}$Radiation-Dominated$\omega_{d}=0$Dust Matter-Dominated
Using Equation (17), we obtained the expression of EoS parameter as
  (29) $$\begin{array}{ccc} \omega_{d} & = & -1-b-\left(\gamma +1\right)\frac{2n}{3}-[\left(\gamma +1\right)\left(72\omega c^{2}-6\left(3\Omega_{d}-12\omega c^{2}\right)\right)(\Omega_{d}(9b+6n\gamma \\ & + & 9+6n)-\left(9+6n\right)\left(\frac{2n}{3}\left(n\omega -3\right)+1\right))][\left(36\Omega_{d}\right)(6+12n-4\omega n^{2}-24\omega c^{2}\\ & + & 2\left(3\Omega_{d}-12\omega c^{2}\right)-24\omega \gamma c^{2}+2\gamma \left(3\Omega_{d}-12\omega c^{2}\right)){]}^{-1}. \end{array}$$
  The equation of state EoS parameter is plotted in the Figure 4 versus redshift *z*. It has been observed that the $\omega_{d}$ lies within the range $-1<\omega_{d}<-\frac{1}{3}$ so it indicates the quintessence era [77].

### 4.2. The BD Theory with a Chameleon Scalar Field

Now, we find the expression of the Hubble parameter, deceleration parameter, jerk parameter and EoS parameter for the BD theory with chameleon scalar field by using the interaction Q1.

**Figure 5:**
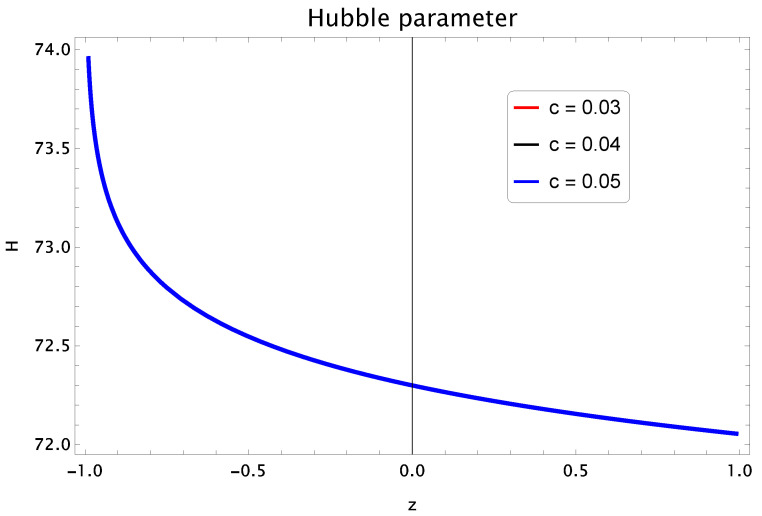
Plot of Hubble parameter *H* versus *z* for interacting KHDE model in the BD theory with a chameleon scalar field.

**Figure 6:**
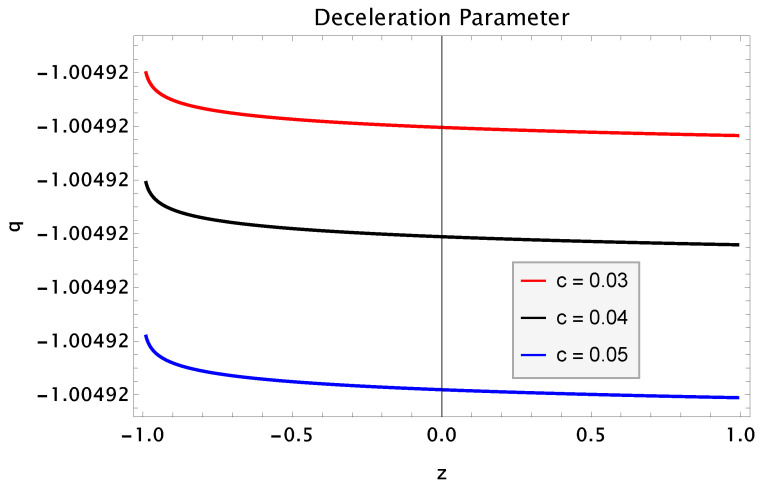
Plot of *q* against *z* for interacting KHDE model in the BD theory with chameleon scalar field.

**Figure 7:**
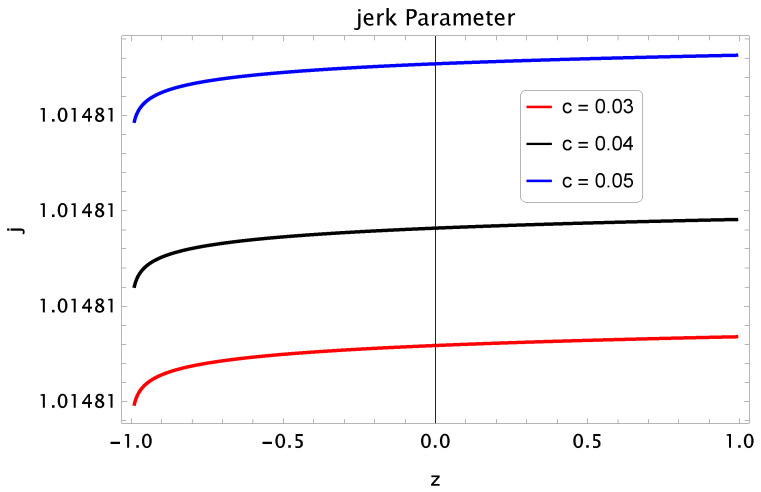
Plot of a jerk parameter *j* against redshift *z* of the KHDE model in the BD theory with a chameleon scalar field.

**Figure 8:**
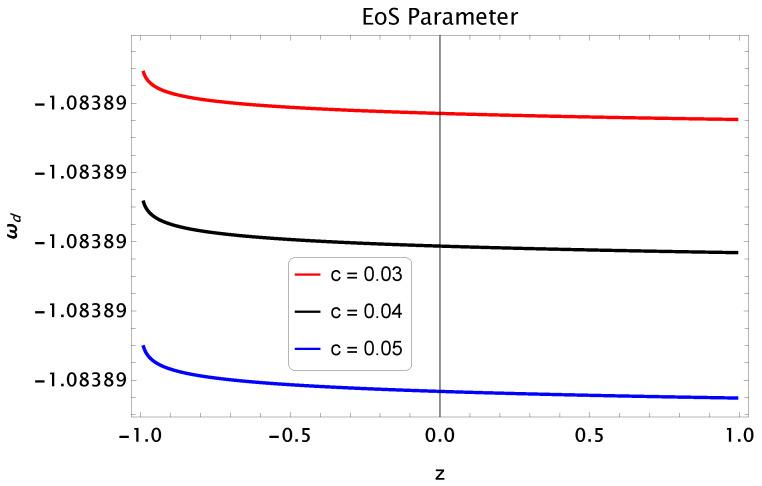
Plot of EoS parameter $\omega_{d}$ against *z* of the KHDE model in the BD theory with a chameleon scalar field for the interacting scenario.

- **Hubble Parameter:** To find the expression of *H*, we use (16) and obtain the following differential equation
  (30) $$\begin{array}{ccc} \frac{\dot{H}}{H^{2}} & = & [12\omega \left(n\gamma -n\right)H^{3}+4\omega^{2}n^{2}H^{3}-4\omega^{2}n^{2}H^{3}\left(n\gamma +n\right)-12n^{2}H^{3}\omega +12n^{2}\omega \gamma H^{3}\\ & + & 4\omega V_{0}\left(n\beta -n\gamma \right)Ha^{n\beta -n}+9f_{0}bH^{3}\Omega_{d}a^{n\gamma +n}-9f_{0}H^{3}a^{n\gamma +n}(\frac{2n}{3}\left(n\omega -3\right)\\ & + & 1-\Omega_{d})+6f_{0}nH^{3}\left(\gamma +1\right)\Omega_{d}a^{n\gamma +n}]\times [24\omega H^{3}-4\omega^{2}n^{2}H^{3}+24n\omega H^{3}\\ & - & 4\omega a^{2n}\left(\gamma +1\right)H^{3}\left(6c^{2}-\frac{3\Omega_{d}-12\omega c^{2}}{2\omega }\right){]}^{-1}. \end{array}$$
  In Figure 5, the Hubble parameter *H* versus the redshift *z* is plotted by selecting the initial value H(z=0)=72.3 for three distinct values of c=0.0002,0.0003,0.0004. Other parameters are b=0.08, $f_{0}=5$, $V_{0}=2$ and β=2. From plot it is clear that the present value of Hubble parameter $H_{0}=72.35$. Also the range of *H* lies in $73_{-1}^{+1}$ for selected value of *z*. It is interesting to mention here that all these values are well matched with observational bounds [77].

- **Deceleration Parameter:** For this theory, the expression of deceleration parameter become
  (31) $$\begin{array}{ccc} q & = & -1-[12\omega \left(n\gamma -n\right)H^{3}+4\omega^{2}n^{2}H^{3}-4\omega^{2}n^{2}H^{3}\left(n\gamma +n\right)-12n^{2}H^{3}\omega +12n^{2}\omega \gamma H^{3}\\ & + & 4\omega V_{0}\left(n\beta -n\gamma \right)Ha^{n\beta -n}+9f_{0}bH^{3}\Omega_{d}a^{n\gamma +n}-9f_{0}H^{3}a^{n\gamma +n}(\frac{2n}{3}\left(n\omega -3\right)+1\\ & - & \Omega_{d})+6f_{0}nH^{3}\left(\gamma +1\right)\Omega_{d}a^{n\gamma +n}]\times [24\omega H^{3}-4\omega^{2}n^{2}H^{3}+24n\omega H^{3}-4\omega a^{2n}\left(\gamma +1\right)\\ & \times & H^{3}\left(6c^{2}-\frac{3\Omega_{d}-12\omega c^{2}}{2\omega }\right){]}^{-1}. \end{array}$$
  The graph of a deceleration parameter *q* is plotted in the Figure 6 along the redshift *z*. It is observed that *q* lies between q<−1 which indicates the expansion is supper exponential in the past, present and later eras.

- **Jerk Parameter:** The expression of the jerk parameter for underlying BD theory with a chameleon scalar field and interaction term yields
  (32) $$\begin{array}{ccc} j & = & (-1-[12\omega \left(n\gamma -n\right)H^{3}+4\omega^{2}n^{2}H^{3}-4\omega^{2}n^{2}H^{3}\left(n\gamma +n\right)-12n^{2}H^{3}\omega \\ & + & 12n^{2}\omega \gamma H^{3}+4\omega V_{0}\left(n\beta -n\gamma \right)Ha^{n\beta -n}+9f_{0}bH^{3}\Omega_{d}a^{n\gamma +n}-9f_{0}H^{3}a^{n\gamma +n}\\ & \times & \left(\frac{2n}{3}\left(n\omega -3\right)+1-\Omega_{d}\right)+6f_{0}nH^{3}\left(\gamma +1\right)\Omega_{d}a^{n\gamma +n}]\times [24\omega H^{3}-4\omega^{2}n^{2}H^{3}\\ & + & 24n\omega H^{3}-4\omega a^{2n}\left(\gamma +1\right)H^{3}\left(6c^{2}-\frac{3\Omega_{d}-12\omega c^{2}}{2\omega }\right){]}^{-1})\left(2q+1\right)+\frac{dq}{dz}\left(1+z\right). \end{array}$$
  The graph of a jerk parameter is shown in Figure 7 against the redshift parameter *z*. For all different values of *c*, we obtained positive behavior of all the trajectories of the jerk parameter that are converges to 1. This showing that the underlying model is compatible with ΛCDM model [77].

- **Equation of State Parameter:** In this scenario, the expression of EoS parameter is defined as
  (33) $$\begin{array}{ccc} \omega_{d} & = & -1-b-\left(\gamma +1\right)\frac{2n}{3}-[\left(\gamma +1\right)\left(72\omega c^{2}-6\left(3\Omega_{d}-12\omega c^{2}\right)\right)(12\omega \left(n\gamma -n\right)H^{3}\\ & + & 4\omega^{2}n^{2}H^{3}-4\omega^{2}n^{2}H^{3}\left(n\gamma +n\right)-12n^{2}H^{3}\omega +12n^{2}\omega \gamma H^{3}+4\omega V_{0}\left(n\beta -n\gamma \right)\\ & \times & Ha^{n\beta -n}+9f_{0}bH^{3}\Omega_{d}a^{n\gamma +n}-9f_{0}H^{3}a^{n\gamma +n}\left(\frac{2n}{3}\left(n\omega -3\right)+1-\Omega_{d}\right)+6f_{0}nH^{3}\\ & \times & \left(\gamma +1\right)\Omega_{d}a^{n\gamma +n})]\times [\left(36\Omega_{d}\right)(24\omega H^{3}-4\omega^{2}n^{2}H^{3}+24n\omega H^{3}-4\omega a^{2n}\left(\gamma +1\right)H^{3}\\ & \times & \left(6c^{2}-\frac{3\Omega_{d}-12\omega c^{2}}{2\omega }\right)){]}^{-1}. \end{array}$$
  In Figure 8 $\omega_{d}$ is plotted against redshift *z* for the three different values of *c*. Since $\omega_{d}$ occurs between $-1<\omega_{d}<-\frac{1}{3}$, it indicates the quintessence era of a universe for all three values of *c* [77].

## 5. $Q_{2}$ Interaction Term

Next, we consider the interaction term $Q_{2}$ and investigate the cosmic evolution of both standard BD and the BD theory with chameleon scalar field theories of gravity. For this interaction term, energy conservation equations become

(34) $$\begin{array}{ccc} {\dot{\rho }}_{d}+3H\rho_{d}\left(1+\omega_{d}\right) & = & -Q_{2}, \end{array}$$

(35) $$\begin{array}{ccc} {\dot{\rho }}_{dm}+3H\rho_{dm} & = & Q_{2}, \end{array}$$

using Equation (), we obtain the expressions of $\rho_{dm}$ and its derivative as

(36) $$\begin{array}{ccc} \rho_{dm} & = & \rho_{m0}\left(a^{\frac{3(1-b)}{\left(\gamma -1\right)}}\right), \end{array}$$

(37) $$\begin{array}{ccc} {\dot{\rho }}_{dm} & = & \rho_{m0}\left(\frac{3(1-b)}{\left(\gamma -1\right)}Ha^{\frac{3(1-b)}{\left(\gamma -1\right)}}\right), \end{array}$$

where $\rho_{m0}$ is the constant of integration.

### 5.1. For Standard BD Theory

Moreover, we find the underlying cosmological parameters for interaction term $Q_{2}$ in the framework of standard BD theory.

**Figure 9:**
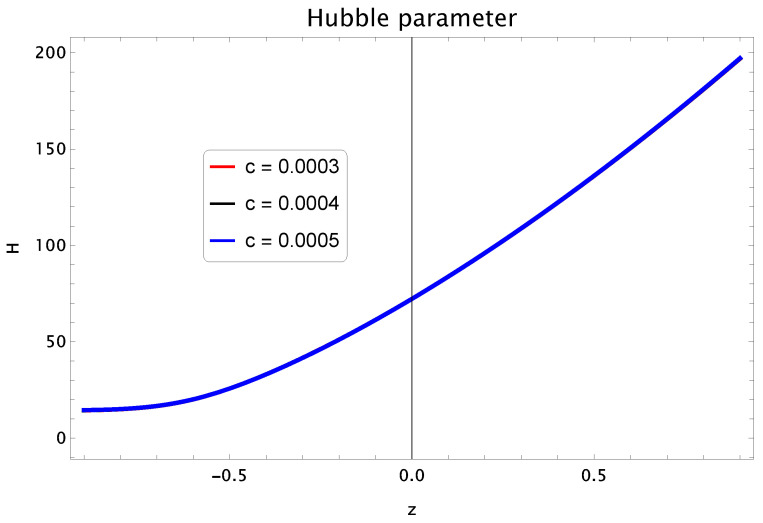
Plot of *H* against *z* for interacting KHDE model in the standard BD theory.

**Figure 10:**
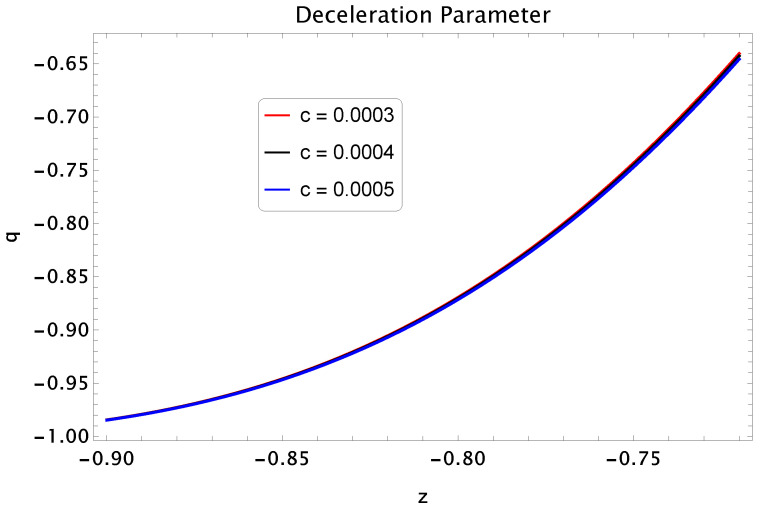
Plot of *q* against *z* for interacting KHDE model in the standard BD theory.

**Figure 11:**
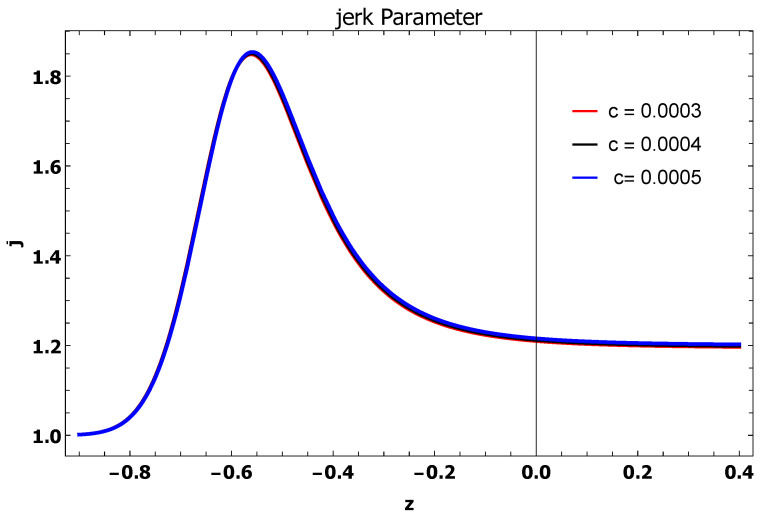
Graph of jerk parameter *j* against *z* for interacting KHDE model in the standard BD theory.

**Figure 12:**
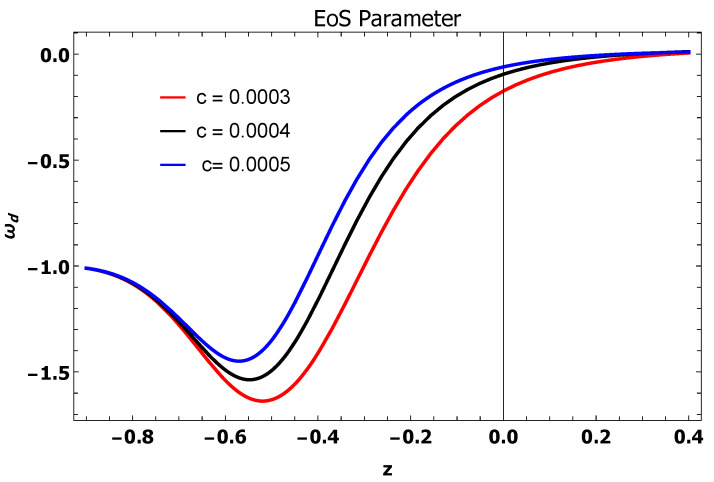
Plot of $\omega_{d}$ against *z* for interacting KHDE model in the standard BD theory.

- **Hubble Parameter:** The expression of *H* for $Q_{2}$ yields
  (38) $$\begin{array}{ccc} \frac{\dot{H}}{H^{2}} & = & [-\Omega_{d}\left(9b-9-6n+9\gamma \left(\frac{1-b}{\gamma -1}\right)\right)+\left(2n\left(n\omega -3\right)+3\right)(3b-3-2n\\ & + & 3\gamma \left(\frac{1-b}{\gamma -1}\right))]\times {\left[6+12n-4\omega n^{2}-24\omega c^{2}+2\left(3\Omega_{d}-12\omega c^{2}\right)\right]}^{-1}. \end{array}$$
  The plot of *H* along *z* is given in Figure 9 by choosing H(z=0)=72.3. We take three different values of c=0.0003,0.0004,0.0005 and b=−0.8. The graph describes that the present value of $H_{0}=75$ which is very near to observational value [77]. Additionally the range of Hubble parameter lies in the range H=105.5±92.5.

- **Deceleration Parameter:** The deceleration parameter for above mention interaction term is
  (39) $$\begin{array}{ccc} q & = & -1-[-\Omega_{d}\left(9b-9-6n+9\gamma \left(\frac{1-b}{\gamma -1}\right)\right)+\left(2n\left(n\omega -3\right)+3\right)(3b-3-2n\\ & + & 3\gamma \left(\frac{1-b}{\gamma -1}\right))]\times {\left[6+12n-4\omega n^{2}-24\omega c^{2}+2\left(3\Omega_{d}-12\omega c^{2}\right)\right]}^{-1}. \end{array}$$
  Figure 10 elaborates the plot of a deceleration parameter versus redshift *z* for three distinct values of *c*. For all values of *c*, *q* lies between −1<q<0 which constitute an accelerated phase of a cosmos [77].

- **Jerk Parameter:** In this case *j* will be
  (40) $$\begin{array}{ccc} j & = & (-1-[-\Omega_{d}\left(9b-9-6n+9\gamma \left(\frac{1-b}{\gamma -1}\right)\right)+\left(2n\left(n\omega -3\right)+3\right)(3b-3-2n\\ & + & 3\gamma \left(\frac{1-b}{\gamma -1}\right))]\times {\left[6+12n-4\omega n^{2}-24\omega c^{2}+2\left(3\Omega_{d}-12\omega c^{2}\right)\right]}^{-1})\times \left(2q+1\right)\\ & + & \frac{dq}{dz}\left(1+z\right). \end{array}$$
  In Figure 11 *j* is plotted along the redshift *z*. It is observed from the graphs that all the trajectories from the past to future era exhibit the positive behavior and converge to 1 which leads to ΛCDM model [77].

- **Equation of State Parameter:** The expression of $\omega_{d}$ of $Q_{2}$ is
  (41) $$\begin{array}{ccc} \omega_{d} & = & -1-\frac{2n}{3}-\left(b+\frac{\gamma (1-b)}{\gamma -1}\right)\left(\frac{\frac{2n}{3}\left(n\omega -3\right)+1-\Omega_{d}}{\Omega_{d}}\right)-[-\Omega_{d}(9b-9-6n\\ & + & 9\gamma \left(\frac{1-b}{\gamma -1}\right))+\left(2n\left(n\omega -3\right)+3\right)\left(3b-3-2n+3\gamma \left(\frac{1-b}{\gamma -1}\right)\right)]\times [(6+12n\\ & - & 4\omega n^{2}-24\omega c^{2}+2\left(3\Omega_{d}-12\omega c^{2}\right))\left(9\Omega_{d}\right){]}^{-1}. \end{array}$$
  The plot of EoS parameter $\omega_{d}$ is given in Figure 12 against the redshift function *z* for different values of *c*. It is shown from the graph that $\omega_{d}$ occurs between $-1<\omega_{d}<-\frac{1}{3}$ which indicates the quintessence era from past to early future when z>−0.2. In the future era, the graph goes quintessence to the phantom era and in the late future approaches to −1 which indicates the cosmological constant. These results favor the observational bounds [77].

### 5.2. For the BD Theory with a Chameleon Scalar Field

Similarly, we calculate the above mentioned cosmological parameters for the BD theory with a chameleon scalar field.

**Figure 13:**
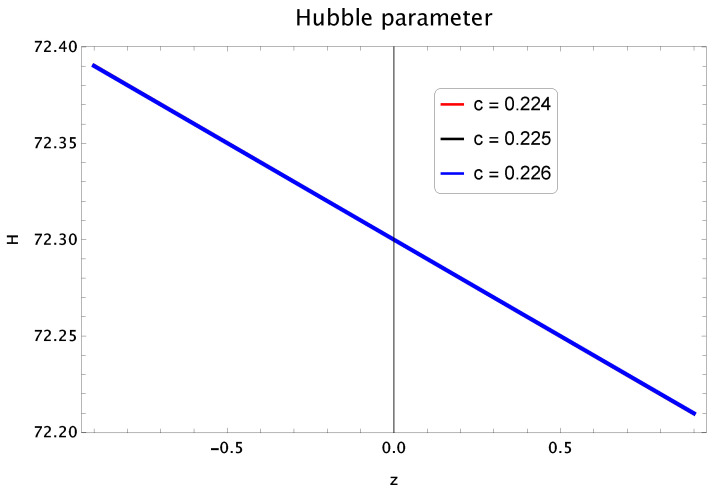
Plot of *H* against *z* for three distinct values of *c* with KHDE model in BD theory with a chameleon scalar field for the interacting scenario.

**Figure 14:**
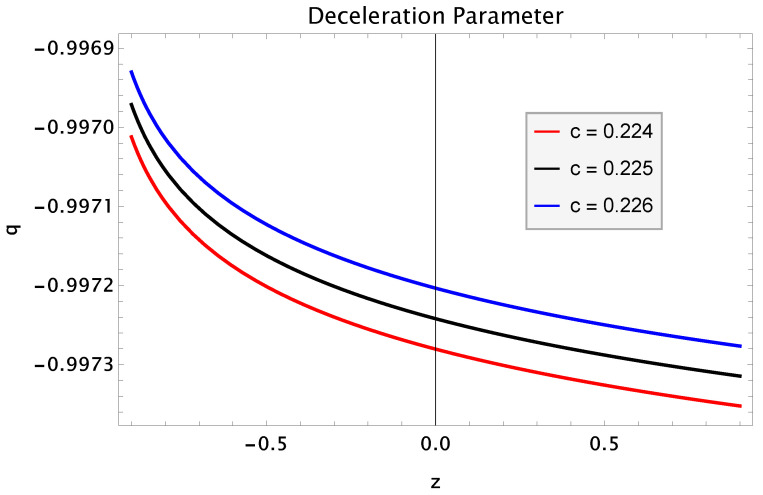
Plot *q* against *z* of the KHDE model in the BD theory with a chameleon scalar field for the interacting scenario.

**Figure 15:**
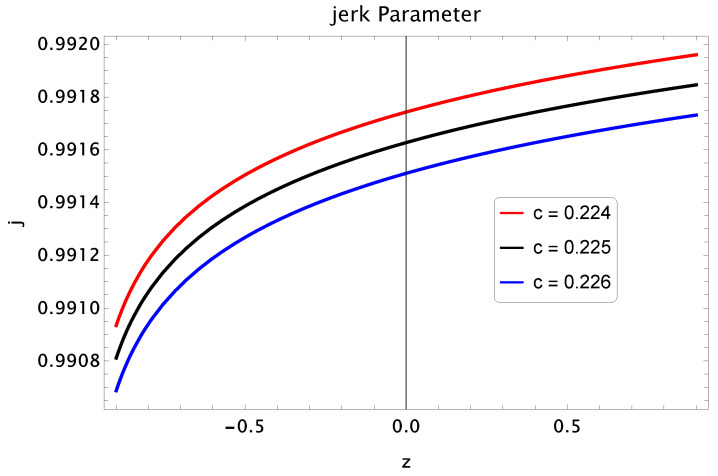
Plot of *j* against *z* for KHDE model in BD theory with a chameleon scalar field for the interacting scenario.

**Figure 16:**
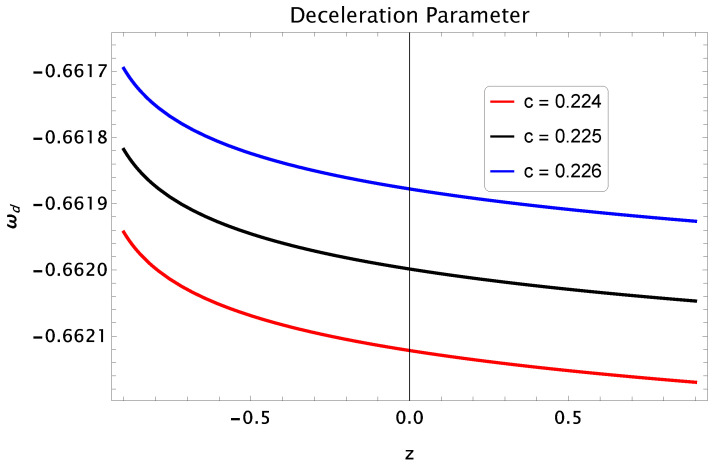
Plot of $\omega_{d}$ against *z* for the KHDE model in BD theory with chameleon scalar field for the interacting scenario.

- **Hubble Parameter:** For this case, the expression of *H* becomes
  (42) $$\begin{array}{ccc} \frac{\dot{H}}{H^{2}} & = & [12\omega \left(n\gamma -n\right)+4\omega n^{3}-2\omega n^{2}\left(n\gamma +n\right)-12\omega n^{2}\left(1+\gamma \right)+4\omega V_{0}\left(n\beta -n\gamma \right)\\ & \times & \frac{a^{n\beta -n}}{H^{2}}+9f_{0}a^{n\gamma +n}\left(b-1+\frac{\gamma (1-b)}{\gamma -1}\right)\left(\frac{2n}{3}\left(n\omega -3\right)+1-\Omega_{d}\right)+6na^{n\gamma +n}\Omega_{d}]\\ & \times & {\left[24\omega -4\omega^{2}n^{2}+24\omega n-\left(6c^{2}-\frac{3\Omega_{d}-12\omega c^{2}}{2\omega }\right)4\omega a^{n\gamma +n}\right]}^{-1}. \end{array}$$
  We plot the Hubble parameter *H* versus *z* in Figure 13 by selecting initial condition H(z=0)=72.3. We take three different values of *c* as c=0.224,0.225,0.226 and b=−0.008. The plot shows that the present value of Hubble parameter $H_{0}=72.30$. Additionally, this parameter *H* lies in the range $H=72.30_{-10}^{+10}$ which shows the consistency with recent observation data [77].

- **Deceleration Parameter:** In this scenario, the expression of *q* is
  (43) $$\begin{array}{ccc} q & = & -1-[12\omega \left(n\gamma -n\right)+4\omega n^{3}-2\omega n^{2}\left(n\gamma +n\right)-12\omega n^{2}\left(1+\gamma \right)+4\omega V_{0}\left(n\beta -n\gamma \right)\\ & \times & \frac{a^{n\beta -n}}{H^{2}}+9f_{0}a^{n\gamma +n}\left(b-1+\frac{\gamma (1-b)}{\gamma -1}\right)\left(\frac{2n}{3}\left(n\omega -3\right)+1-\Omega_{d}\right)+6na^{n\gamma +n}\Omega_{d}]\\ & \times & {\left[24\omega -4\omega^{2}n^{2}+24\omega n-\left(6c^{2}-\frac{3\Omega_{d}-12\omega c^{2}}{2\omega }\right)4\omega a^{n\gamma +n}\right]}^{-1}. \end{array}$$
  In Figure 14, the deceleration parameter *q* is plotted against redshift *z*. It is observed from the plot that all the trajectories from past to future lie between −1<q<0 which corresponds to very fast accelerated expansion of the universe (power law expansion).

- **Jerk Parameter:** In BD theory with a chameleon scalar field, the expression of *j* is
  (44) $$\begin{array}{ccc} j & = & (-1-[12\omega \left(n\gamma -n\right)+4\omega n^{3}-2\omega n^{2}\left(n\gamma +n\right)-12\omega n^{2}\left(1+\gamma \right)+4\omega V_{0}(n\beta \\ & - & n\gamma )\frac{a^{n\beta -n}}{H^{2}}+9f_{0}a^{n\gamma +n}\left(b-1+\frac{\gamma (1-b)}{\gamma -1}\right)\left(\frac{2n}{3}\left(n\omega -3\right)+1-\Omega_{d}\right)+6na^{n\gamma +n}\Omega_{d}]\\ & \times & {\left[24\omega -4\omega^{2}n^{2}+24\omega n-\left(6c^{2}-\frac{3\Omega_{d}-12\omega c^{2}}{2\omega }\right)4\omega a^{n\gamma +n}\right]}^{-1})\times \left(2q+1\right)\\ & + & \frac{dq}{dz}\left(1+z\right). \end{array}$$
  The jerk parameter *j* is plotted in Figure 15 versus *z*. It can be seen from the graph that all trajectories give the positive behavior for all cases of *c*. In all cases, trajectories favor the ΛCDM limit.

- **Equation of State Parameter:** The expression of $\omega_{d}$ for this case will be
  (45) $$\begin{array}{ccc} \omega_{d} & = & -1-\frac{2n}{3}-\left(b+\frac{\gamma (1-b)}{\gamma -1}\right)\left(\frac{\frac{2n}{3}\left(n\omega -3\right)+1-\Omega_{d}}{\Omega_{d}}\right)-[(24\omega c^{2}-2(3\Omega_{d}-12\omega \\ & \times & c^{2}))(12\omega \left(n\gamma -n\right)+4\omega n^{3}-2\omega n^{2}\left(n\gamma +n\right)-12\omega n^{2}\left(1+\gamma \right)+4\omega V_{0}\left(n\beta -n\gamma \right)\\ & \times & \frac{a^{n\beta -n}}{H^{2}}+9f_{0}a^{n\gamma +n}\left(b-1+\frac{\gamma (1-b)}{\gamma -1}\right)\left(\frac{2n}{3}\left(n\omega -3\right)+1-\Omega_{d}\right)+6na^{n\gamma +n}\Omega_{d})]\\ & \times & [\left(36\omega c^{2}+3\left(3\Omega_{d}-12\omega c^{2}\right)\right)(24\omega -4\omega^{2}n^{2}+24\omega n-\left(6c^{2}-\frac{3\Omega_{d}-12\omega c^{2}}{2\omega }\right)\\ & \times & 4\omega a^{n\gamma +n}){]}^{-1}. \end{array}$$
  The plot of EoS $\omega_{d}$ against redshift function *z* is given in Figure 16. It is observed from the graph that $\omega_{d}$ lies between $-1<\omega_{d}<-\frac{1}{3}$ which indicates the quintessence era of the universe. It is interesting to mention here that these results are compatible with observational data [77].

## 6. Thermodynamics

Researchers examined the thermodynamical properties of various DE models of the cosmos in the framework of a rapid universe expansion. These properties are driven by the study of black hole physics and discovered a strong relationship between gravity and thermodynamics [81,82,83]. According to thermodynamics, the rate of change in entropy and heat capacity must be positive for an accelerated expansion of the universe. Thus, thermodynamical axioms have become the key factor to check the stability of the model. The thermodynamic and fluid-dynamic features of gravity provide us with other perspectives to comprehend gravity in the absence of a quantum theory. Thermodynamics is a physical science that is concerned with the concepts of energy, heat and temperature as well as how they relate to radiation, energy, and the physical properties of matter. When we consider observable macroscopic physical quantities like volume, pressure, internal energy, temperature and entropy, the four thermodynamic properties provide a significant explanation for the behavior of these values. In these properties, the generalized second law of thermodynamics (GSLT) is very important. This law is defined as the total entropy of an isolated system must be non-negative $\left({\dot{S}}_{tot}\right)={\dot{S}}_{u}+{\dot{S}}_{int}\geq 0$ (where ${\dot{S}}_{int},\;{\dot{S}}_{u}$ are the rate of change of the internal and external entropies of the system). To check the validity of GSLT, first we start with the Gibbs equation [84], in which the entropy of the universe inside the horizon is represented by its energy and pressure as

(46) $$\begin{array}{c} TdS_{int}=dE+pdV, \end{array}$$

that can be rewritten in following expression

(47) $$\begin{array}{c} {\dot{S}}_{int}=\frac{1}{T}\left(\dot{E}+p\dot{V}\right), \end{array}$$

here *V* represents the volume which defined as $V=\frac{4\pi R_{A}^{3}}{3}$ (for flat FRW universe $R_{A}=\frac{1}{H}$), *E* is the total energy of the system that is E=ρV and *T* is the fluid’s temperature which bounded by the horizon. To avoid non-equilibrium thermodynamic’s mathematical complexity in that case. We also assume that there is no sudden energy exchange between the fluid and the horizon. Therefore, it assumed that the cosmological fluid *T* within a horizon is at the same degree as the bounded horizon $T_{h}$ that is $T=T_{h}$. Now, we take derivatives of *V*, *E* w.r.t time and then put these in Equation (47), we obtain

(48) $$\begin{array}{c} {\dot{S}}_{int}=\frac{8\pi^{2}}{H^{4}}\left[\frac{\dot{\rho }}{3}-\left(\rho +p\right)\frac{\dot{H}}{H}\right]. \end{array}$$

For external scenarios, the combination of Kaniadakis and Bekenstein Hawking entropies is given as [54,55]

(49) $$\begin{array}{c} S_{u}=S_{BH}+\frac{u^{2}}{6}S_{BH}^{3}. \end{array}$$

By taking derivative of Equation (49) w.r.t time we get

(50) $$\begin{array}{c} {\dot{S}}_{u}=-\frac{2\pi \dot{H}}{GH^{3}}-\frac{u^{2}\dot{H}}{G^{3}H^{7}}, \end{array}$$

where $S_{BH}=\frac{A}{4G}$ and $A=4\pi R_{A}^{2}$. Using Equation (48) and (50), the total entropy of the system become

(51) $$\begin{array}{c} {\dot{S}}_{tot}=\frac{8\pi^{2}}{H^{4}}\left[\frac{\dot{\rho }}{3}-\left(\rho +p\right)\frac{\dot{H}}{H}\right]-\left(\frac{2\pi }{GH^{2}}+\frac{u^{2}}{G^{3}H^{6}}\right)\frac{\dot{H}}{H}. \end{array}$$

### 6.1. For Standard Brans–Dicke Theory of Gravity

For interaction $Q_{1}$, we obtain the expression of total entropy $\frac{dS}{da}$ by using Equation (51) as the function of scale factor as

(52) $$\begin{array}{ccc} \frac{dS}{da} & = & -[\left(\Omega_{d}\left(9b+6n\gamma +9+6n\right)-\left(9+6n\right)\left(\frac{2n(n\omega -3)}{3}+1\right)\right)(\frac{6\pi^{2}a^{2n}\left(1+\omega \right)}{\omega H^{2}}\\ & \times & \left(\frac{2n(n\omega -3)}{3}+1\right)+\frac{2\pi }{GH^{2}}+\frac{u^{2}}{G^{3}H^{6}}-\left(\left(\frac{8\pi^{2}a^{2n}}{3H^{2}}\right)\left(6c^{2}-\frac{2u^{2}}{H^{4}}\right)\right)\left(\gamma +1\right))]\\ & \times & {\left[\left(a\right)\left(6+12n-4\omega n^{2}-48\omega c^{2}+6\Omega_{d}-24\gamma \omega c^{2}+\gamma \left(6\Omega_{d}-24\omega c^{2}\right)\right)\right]}^{-1}+\frac{8\pi^{2}}{3H^{2}a}\\ & \times & \left[\frac{a^{2n}\Omega_{d}}{4\omega }\left(9b+6n\gamma +9+6n\right)-\frac{9a^{2n}}{4\omega }\left(\frac{2n(n\omega -3)}{3}+1\right)\right]. \end{array}$$

**Figure 17:**
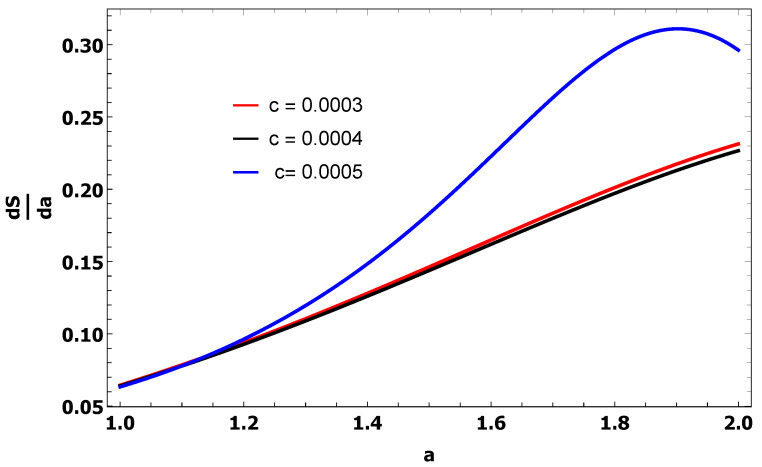
Plot of $\frac{ds}{da}$ against *a* of the interacting KHDE model in the standard BD theory.

The plot of the total entropy $\frac{dS}{da}$ versus *a* by selecting initial value H(a=1)=72.3 is shown in Figure 17. It can be observed from the graph that $\frac{dS}{da}>0$ for all the trajectories which indicates that the GSLT is valid for the KHDE model in the framework of standard BD theory. Similarly, for interaction $Q_{2}$, the expression of $\frac{dS}{da}$ will be

(53) $$\begin{array}{ccc} \frac{dS}{da} & = & -[(-\Omega_{d}\left(9b-9-6n+9\gamma \left(\frac{1-b}{\gamma -1}\right)\right)+\left(2n\left(n\omega -3\right)+3\right)(3b-3-2n+3\gamma \\ & \times & \left(\frac{1-b}{\gamma -1}\right)))\times (\frac{6\pi^{2}a^{2n}\left(1+\omega \right)}{\omega H^{2}}\left(\frac{2n(n\omega -3)}{3}+1\right)+\frac{2\pi }{GH^{2}}+\frac{u^{2}}{G^{3}H^{6}}-\frac{8\pi^{2}a^{2n}}{3H^{2}}(6c^{2}\\ & - & \frac{2u^{2}}{H^{4}}))]{\left[\left(a\right)\left(6+12n-4\omega n^{2}-24\omega c^{2}+2\left(3\Omega_{d}-12\omega c^{2}\right)\right)\right]}^{-1}+\frac{8\pi^{2}}{3H^{2}a}(\frac{a^{2n}}{4\omega }\Omega_{d}\\ & \times & \left(-9b-\frac{9\gamma (1-b)}{\gamma -1}+9+6n\right)+\frac{9a^{2n}}{4\omega }\left(\frac{2n}{3}\left(n\omega -3\right)+1\right)\left(b+\frac{\gamma (1-b)}{\gamma -1}-1\right)). \end{array}$$

**Figure 18:**
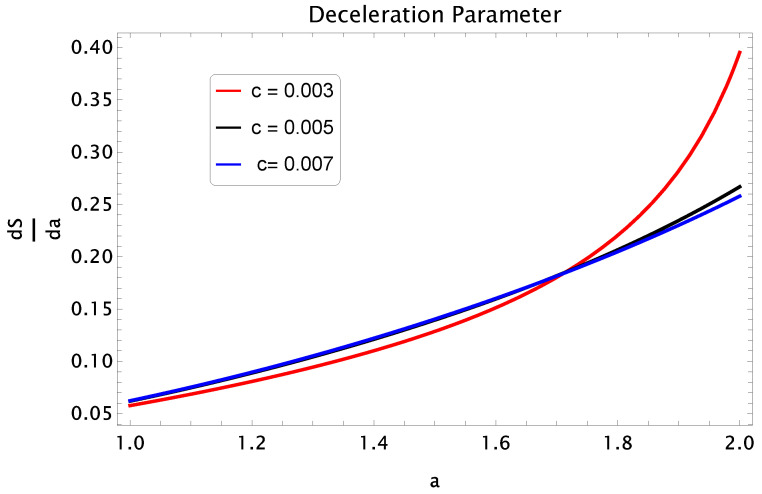
Plot of an entropy $\frac{ds}{da}$ against *a* for the interacting KHDE in the standard BD theory.

In Figure 18, total entropy $\frac{dS}{da}$ is plotted against the scale factor *a* by choosing initial value H(a=1)=72.3. We can see that all trajectories are positive for all selected values of *c* which provide the validity of GSLT.

### 6.2. For the BD Theory with a Chameleon Scalar Field

Now, we consider the interaction $Q_{1}$ for the BD theory with a chameleon scalar field. For this choice we obtain $\frac{dS}{da}$ as follows

(54) $$\begin{array}{ccc} \frac{dS}{da} & = & -[(12\omega \left(n\gamma -n\right)H^{3}+4\omega^{2}n^{2}H^{3}-4\omega^{2}n^{2}H^{3}\left(n\gamma +n\right)-12n^{2}H^{3}\omega +12n^{2}\omega \gamma H^{3}\\ & + & 4\omega V_{0}\left(n\beta -n\gamma \right)Ha^{n\beta -n}+9f_{0}bH^{3}\Omega_{d}a^{n\gamma +n}-9f_{0}H^{3}a^{n\gamma +n}\left(\frac{2n}{3}\left(n\omega -3\right)+1-\Omega_{d}\right)\\ & + & 6f_{0}nH^{3}\left(\gamma +1\right)\Omega_{d}a^{n\gamma +n})(\frac{6\pi^{2}a^{2n}\left(1+\omega \right)}{\omega H^{2}}\left(\frac{2n(n\omega -3)}{3}+1\right)+\frac{2\pi }{GH^{2}}+\frac{u^{2}}{G^{3}H^{6}}\\ & - & \left(\left(\frac{8\pi^{2}a^{2n}}{3H^{2}}\right)\left(6c^{2}-\frac{2u^{2}}{H^{4}}\right)\right)\left(\gamma +1\right))]\times [\left(a\right)(24\omega H^{3}-4\omega^{2}n^{2}H^{3}+24n\omega H^{3}-4\omega a^{2n}\\ & \times & \left(\gamma +1\right)H^{3}\left(6c^{2}-\frac{3\Omega_{d}-12\omega c^{2}}{2\omega }\right)){]}^{-1}+\frac{8\pi^{2}}{3aH^{2}}[\frac{a^{2n}\Omega_{d}}{4\omega }\left(9b+6n\gamma +9+6n\right)-\frac{9a^{2n}}{4\omega }\\ & \times & \left(\frac{2n(n\omega -3)}{3}+1\right)]. \end{array}$$

**Figure 19:**
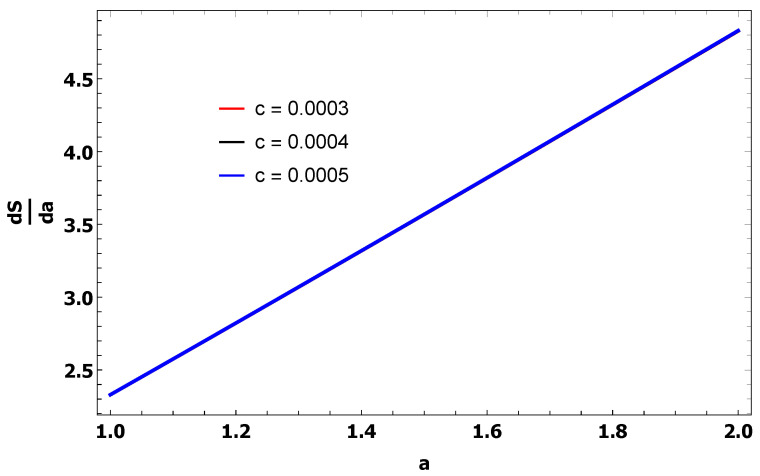
Plot of an entropy $\frac{ds}{da}$ against *a* of a KHDE in the BD theory with a chameleon scalar field for the interacting scenario $Q_{1}$.

Figure 19 represents the plot of the total entropy $\frac{dS}{da}$ versus the scale factor *a* by selecting H(a=1)=72.3. It is clear from the graph that the GSLT is valid for the KHDE in the BD theory with a chameleon scalar field, because all the trajectories are positive for three different values of *c*. Again, by using the $Q_{2}$ interaction, we obtain the expression of $\frac{dS}{da}$ for the BD theory with a chameleon scalar field as follows:

(55) $$\begin{array}{ccc} \frac{dS}{da} & = & -[(12\omega \left(n\gamma -n\right)+4\omega n^{3}-2\omega n^{2}\left(n\gamma +n\right)-12\omega n^{2}\left(1+\gamma \right)+4\omega V_{0}\left(n\beta -n\gamma \right)\frac{a^{n\beta -n}}{H^{2}}\\ & + & 9f_{0}a^{n\gamma +n}\left(b-1+\frac{\gamma (1-b)}{\gamma -1}\right)\left(\frac{2n}{3}\left(n\omega -3\right)+1-\Omega_{d}\right)+6na^{n\gamma +n}\Omega_{d})(\frac{6\pi^{2}a^{2n}\left(1+\omega \right)}{\omega H^{2}}\\ & \times & \left(\frac{2n(n\omega -3)}{3}+1\right)+\frac{2\pi }{GH^{2}}+\frac{u^{2}}{G^{3}H^{6}}-\frac{8\pi^{2}a^{2n}}{3H^{2}}\left(6c^{2}-\frac{2u^{2}}{H^{4}}\right))][\left(a\right)(24\omega -4\omega^{2}n^{2}\\ & + & 24\omega n-\left(6c^{2}-\frac{3\Omega_{d}-12\omega c^{2}}{2\omega }\right)4\omega a^{n\gamma +n}){]}^{-1}+\frac{8\pi^{2}}{3aH^{2}}(\frac{a^{2n}}{4\omega }\Omega_{d}(-9b-\frac{9\gamma (1-b)}{\gamma -1}\\ & + & 9+6n)+\frac{9a^{2n}}{4\omega }\left(\frac{2n}{3}\left(n\omega -3\right)+1\right)\left(b+\frac{\gamma (1-b)}{\gamma -1}-1\right)). \end{array}$$

**Figure 20:**
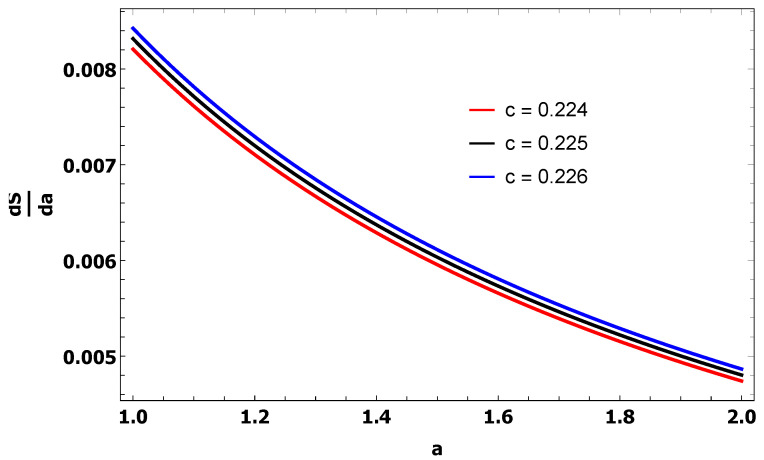
Plot of an entropy $\frac{ds}{da}$ against *a* for an interacting KHDE model in the BD theory with the chameleon scalar field.

The plot of total entropy $\frac{dS}{da}$ is plotted in Figure 20 against *a* by choosing the initial value H(a=1)=72.3. All the curves showed positive behavior for three different values of *c*, which gives the validity of the GSLT.

## 7. Conclusions and Comparison

Yang et al. [85] studied the correspondence between the tachyon, quintessence, dilaton scalar field, K-essence, and Chaplygin gas model with the non-interacting new HDE model in the non-flat BD theory. They reconstructed the potentials and dynamics for these models and found the accelerated expansion of the universe in the context of the BD theory. In Ref. [66], the authors reconstructed a new HDE model with $\phi =\phi_{0}a^{\alpha }\;V=V_{0}\phi^{\beta }$ and $f=f_{0}\phi^{\gamma }$. They considered the chameleon BD cosmology and worked on the correspondence between the quintessence, the DBI-essence, and the tachyon scalar field models with the non-interacting new HDE model. They also found the expression of the Hubble parameter and stability for the obtained solutions of the crossing of the phantom divide. They found that the potential increases as the matter-chameleon coupling gets stronger with the evolution of the universe.

The basic purpose of this paper was to explore the effects of interaction terms on the cosmology of KHDE in the context of BD and the BD theory with a chameleon scalar field for the flat FRW universe. We have investigated the cosmographical parameters, such as Hubble parameter *H*, the deceleration parameter *q*, the EoS parameter $\omega_{d}$, and the jerk parameter *j* in the framework of BD and the BD theory with a chameleon scalar field for the flat FRW universe. We also discussed the GSLT of KHDE for the standard BD and the BD theory with a chameleon scalar field. We take the values of constant parameters for both interaction terms in standard BD theory as u=−0.9,γ=−0.8,n=0.0009. The values of constants in the BD theory with a chameleon scalar field for $Q_{1}$ term are taken as u=0.01,γ=0.002,n=0.0009, and for the $Q_{2}$ term as u=−0.09,γ=−0.7,andn=0.09. Throughout the paper, the Brans–Dicke coupling constant is assumed to be ω= 40,000 [86].

We observed the effects of interaction term $Q_{1}$ in the context of the standard BD theory on the Hubble parameter *H*, which have shown the positive behavior that represents the universe expanded, that *q* has gone from a decelerated phase to an accelerated phase shown and that the expansion of the universe is accelerated, the jerk parameter *j* has shown positive behavior, and EoS $\omega_{d}$ is in the quintessence era. It has been found that the effects of interaction term $Q_{1}$ in the context of BD theory with a chameleon scalar field on the Hubble parameter and the deceleration parameter gave us an accelerated expansion (see Figure 5 and Figure 6). Meanwhile, the jerk parameter *j* has shown positive behavior for all the trajectories, and $\omega_{d}$ indicated the quintessence era.

We examined the effects of the $Q_{2}$ interaction term in the framework of standard BD theory. It has been found that the Hubble parameter *H* and the deceleration parameter *q* show the accelerated expansion of the universe. The jerk parameter indicated positive behavior and converged to −1 when z⟶−1, and the EoS parameter varies from quintessence to the phantom region and approaches to −1. It has been observed that the effects of interaction term $Q_{2}$ are in the context of BD theory with a chameleon scalar field. Here, we observed that the Hubble parameter *H*, as well as the deceleration parameter *q*, shows the accelerated expansion of the universe. For this interaction term, the trajectories of the jerk parameter and EoS parameter provide us the universe’s quintessence phase. We also contrasted our results with observational data related to the EoS parameter. We observed that the EoS parameter results are compatible with those from the Planck collaboration [77]. The following are the observational data:
$\omega_{d}$Observational Schemes${-1.56}_{-0.48}^{+0.60}$Planck+TT+lowE${-1.58}_{-0.41}^{+0.52}$Planck+TT, TE, EE+lowE${-1.57}_{-0.40}^{+0.50}$Planck+TT, TE, EE+lowE+lensing${-1.40}_{-0.10}^{+0.10}$Planck+TT, TE, EE+lowE+lensing+BAO

We also examined the results of deceleration parameter *q* with observational data [87], which demonstrates its compatible behavior. The following are the observational data for *q*:
deceleration parameterObservational Schemes−0.644∓0.223BAO+Masers+TDSL+Pantheon−0.6401∓0.187BAO+Masers+TDSL+Pantheon+$H_{0}$−0.930∓0.218BAO+Masers+TDSL+Pantheon+$H_{z}$−1.2037∓0.175BAO+Masers+TDSL+Pantheon+$H_{0}$ +$H_{z}$

We also observed that the GSLT for the KHDE in the Standard BD theory, as well as the BD theory with a chameleon scalar field, shows increasing behavior $\left(\frac{dS}{da}>0\right)$ for both interaction terms $Q_{1}$ and $Q_{2}$, which ensures that the GSLT is valid for the selected range of scale factor. 

## Data Availability

There is no other asscoiated data related to this manuscript.
